# Prognostic and clinicopathological value of fibrinogen-to-albumin ratio in colorectal cancer: a meta-analysis

**DOI:** 10.1080/07853890.2025.2530689

**Published:** 2025-07-11

**Authors:** Yanguang Su, Juanli Chen, Lingjun Du, Xudong Liu

**Affiliations:** Operating Room, Huzhou Central Hospital, Affiliated Central Hospital of Huzhou University, Huzhou, Zhejiang, China

**Keywords:** Biomarker, colorectal cancer, evidence-based medicine, fibrinogen-to-albumin ratio, prognosis

## Abstract

**Background:**

The fibrinogen-to-albumin ratio (FAR) has been extensively studied for its potential to predict the prognosis of patients with colorectal cancer (CRC). However, findings have been inconsistent. Therefore, this meta-analysis aims to examine the prognostic value of FAR in CRC.

**Methods:**

A comprehensive search of PubMed, Web of Science, Cochrane Library, and Embase was conducted up to January 14, 2025. Hazard ratios (HRs) and 95% confidence intervals (CIs) were calculated to assess the value of FAR for estimating overall survival (OS) and progression-free survival (PFS) in patients with CRC. Additionally, the relationship between FAR and CRC clinicopathological characteristics was explored using pooled odds ratios (ORs) with corresponding 95% CIs.

**Results:**

This meta-analysis included 10 studies comprising 4,704 patients. The findings indicated that elevated FAR was significantly associated with worse OS (HR = 1.59, 95% CI = 1.38–1.83, *p* < 0.001) and PFS (HR = 1.65, 95% CI = 1.44–1.90, *p* < 0.001) among patients with CRC. Subgroup analyses confirmed that high FAR consistently predicted unfavorable OS and PFS, regardless of study design, histology, treatment, FAR threshold, threshold determination method, or type of survival analysis (all *p* < 0.05). Moreover, elevated FAR was significantly associated with age ≥60 years (OR = 1.56, 95% CI = 1.31–1.85, *p* < 0.001), male sex (OR = 1.20, 95% CI = 1.01–1.43, *p* = 0.042), and poor tumor differentiation (OR = 1.63, 95% CI = 1.26–2.10, *p* < 0.001).

**Conclusions:**

Elevated FAR is strongly associated with poor OS and PFS in patients with CRC, as well as with older age and poor tumor differentiation, suggesting its potential as a prognostic marker.

## KEY MESSAGES

1. To our knowledge, this meta-analysis has first investigated FAR’s value for predicting CRC prognosis.
2. Higher FAR is significantly linked with poor OS and PFS for CRC cases.
3. Higher FAR is also significantly connected to elderly age and poor differentiation in CRC.

## Introduction

Colorectal cancer (CRC) is the third most common cancer worldwide and the second leading cause of cancer-related mortality [1]. Notably, cancers of unknown primary site (CUP) account for approximately 1–3% of all malignant tumors [2]. Among these, CUP with a colon cancer profile is increasingly recognized as a distinct favorable subgroup [2]. According to GLOBOCAN data, 1,880,725 new cases of CRC and 615,880 deaths attributed to the disease were recorded globally in 2020 [3]. Advanced or intermediate-stage CRC is often associated with hematogenous metastasis to the liver, which significantly worsens prognosis and increases the risk of mortality [4]. Despite advancements in multimodal treatment approaches and tailored chemoradiotherapy, achieving long-term survival and effective disease management remains a significant challenge for patients with metastatic CRC [5]. Notably, the risk of CRC increases with age, with over two-thirds of cases occurring in individuals aged 65 years and older [6]. Although older patients are at higher risk for severe postoperative complications, no consensus exists regarding the impact of age on survival outcomes [7]. The prognosis for older patients might be affected by factors such as the stage of disease at diagnosis, tumor location, preexisting health conditions, and treatment modalities[8]. Although immunotherapy has recently emerged as a treatment option for patients with CRC, the prognosis for advanced and metastatic CRC cases remains poor [9,10]. Programmed cell death-ligand 1 (PD-L1) expression in immune cells is significantly elevated in mismatch repair (MMR)-deficient (MSI-H) CRC compared to MMR-proficient (MSI-L) tumors, with consistent expression across various MSI-H molecular subtypes [9]. Screening for defective DNA MMR using immunohistochemistry (IHC) and/or microsatellite instability testing is recommended, though converting the biological and technical variability of microsatellite instability tests into practical clinical data remains a challenge [11]. Research suggests that IHC testing of the MMR may produce inconsistent outcomes for specific germline mutations, potentially due to accompanying somatic mutations [12,13]. The 5-year survival rate for stage IIIC CRC is 53%, whereas for metastatic CRC, it drops to only 12% [10]. Biomarkers play a crucial role in guiding CRC therapy and improving survival rates [14]. Consequently, biomarker testing is recommended as a part of the standard diagnostic process for CRC [1]. The RAS mutation, a key genetic alteration in CRC, is associated with increased tumor aggressiveness and resistance to chemotherapy [14]. Moreover, microRNAs (miRNAs) function as both tumor suppressors and oncogenes, and their diagnostic, prognostic, and predictive potential is currently under investigation [13,14]. For instance, in metastatic CRC, resistance to anti-vascular endothelial growth factor or anti-epidermal growth factor receptor inhibitors has been linked to specific miRNA expression patterns. An increase in miR-126 is associated with resistance to bevacizumab, while resistance to cetuximab is observed with overexpression of miR-31, miR-100, and miR-125b, as well as downregulation of miR-7 [1,14]. Therefore, an urgent need exists to identify novel prognostic markers to enhance the clinical management of CRC.

Emerging evidence highlights the critical roles of nutrition and inflammation in CRC metastasis and progression [12,15]. Various inflammatory parameters, such as platelet-to-lymphocyte ratio [16], prognostic nutritional index [17], lymphocyte-to-monocyte ratio [18], controlling nutritional status score [19], and geriatric nutritional risk index[20], have been identified as prognostic markers for CRC. The fibrinogen–albumin ratio (FAR) is a novel inflammatory and nutritional biomarker that has demonstrated prognostic efficiency across diverse cancers, including esophageal cancer [21], hepatocellular carcinoma [22], osteosarcoma [23], non-small-cell lung cancer [24], and ovarian cancer [25]. Although recent studies have explored FAR’s potential in predicting CRC prognosis, no consistent findings have been obtained [26–35]. Some studies report that a higher FAR is significantly associated with poor CRC prognosis [27,29,34], whereas others found no significant correlation between FAR and CRC survival [30,33]. Therefore, this meta-analysis aimed to identify the precise role of FAR in predicting CRC prognosis and explore its relationship with clinicopathological factors in CRC.

## Materials and methods

### Study guideline

This meta-analysis was conducted in accordance with the Preferred Reporting Items for Systematic reviews and Meta-Analyses (PRISMA) guideline [36].

### Ethics statement

As this study is a meta-analysis of previously published research, it did not require ethical approval.

### Literature search

A comprehensive search of PubMed, Web of Science, Cochrane Library, and Embase databases was conducted up to January 14, 2025. The search strategy included the terms (albumin-to-fibrinogen OR albumin/fibrinogen OR fibrinogen-to-albumin OR fibrinogen/albumin OR Alb to Fib) AND (colonic neoplasms OR colon cancer OR colorectal neoplasms OR rectal cancer OR rectal tumor OR rectum cancers OR colorectal cancer OR CRC OR colorectal carcinoma OR colorectal tumor). Both Medical Subject Headings terms and free-text keywords were utilized. Only studies published in English were included in this meta-analysis. Additionally, the reference lists of identified articles were reviewed to identify potentially relevant studies.

### Inclusion and exclusion criteria

The inclusion criteria were as follows: (1) studies involving patients with a pathological diagnosis of CRC; (2) studies that examined the relationship between FAR and clinical outcomes in CRC cases; (3) studies with extractable or computable hazard ratios (HRs) and 95% confidence intervals (CIs); (4) available FAR thresholds; and (5) studies published in English. The exclusion criteria were: (1) letters, reviews, comments, and conference abstracts; (2) studies with insufficient data for meta-analysis; and (3) animal studies.

### Data extraction and quality assessment

Two investigators (Y.S. and J.C.) independently screened eligible studies and extracted data. Disagreements were resolved through discussion with a third reviewer (X.L.) until a consensus was reached. Extracted data included first author, publication year, country, sample size, sex, age, study period, study design, histology, TNM stage, treatment details, FAR threshold, threshold determination method, survival endpoints, survival analysis types, follow-up duration, HRs, and 95% CIs. Overall survival (OS) and progression-free survival (PFS) were the primary and secondary endpoints, respectively. The quality of the included studies was assessed using the Newcastle–Ottawa Scale (NOS), which covers three domains: selection, comparability, and outcome assessment [37]. NOS scores range from 0 to 9 points, and studies scoring ≥6 points were considered high-quality.

### Statistical analysis

In this study, pooled HRs and 95% CIs were computed to evaluate the prognostic value of FAR for predicting OS and PFS in CRC. Inter-study heterogeneity was assessed using Cochran’s *Q* test and *I*^2^ statistics. A threshold of *p* < 0.10 and *I*^2^ > 50% indicated significant heterogeneity, in which case a random-effects model was used; otherwise, a fixed-effects model was applied. Subgroup analyses were also conducted. The association between FAR and clinicopathological factors in CRC was explored using pooled odds ratios (ORs) and 95% CIs. Additionally, sensitivity analysis was performed by sequentially omitting one article at a time to assess the robustness of our results. Publication bias was evaluated using Begg’s and Egger’s tests. Statistical analysis was performed using Stata version 12.0 software (Stata Corporation, College Station, TX, USA), with statistical significance set at *p* < 0.05.

## Results

### Literature retrieval process

**Figure 1.**
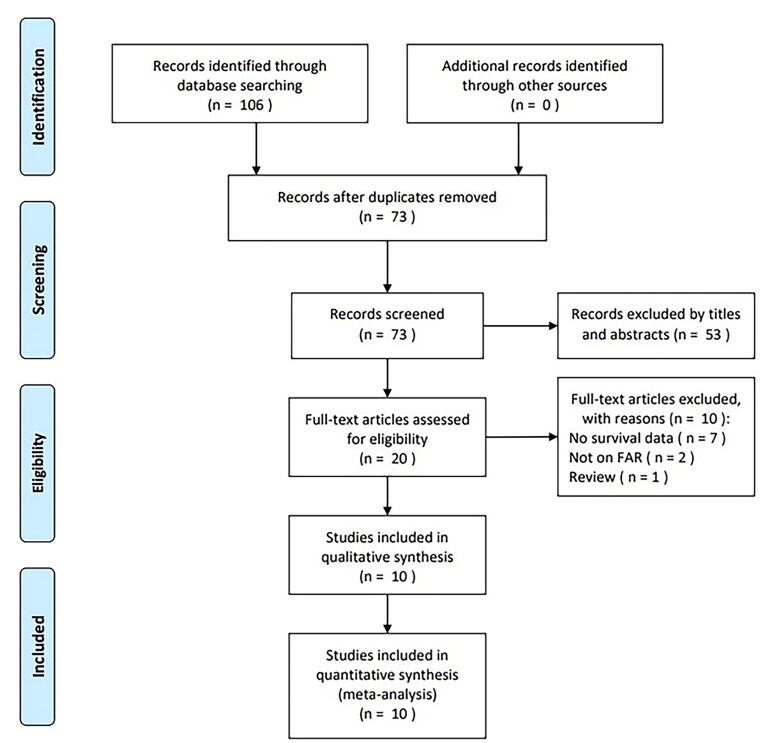
PRISMA flowchart of the literature search.

The initial literature search identified 106 studies, with 73 remaining after duplicates were removed (Figure 1). After title and abstract screening, 53 studies were excluded due to irrelevance or being animal studies. Twenty articles were further assessed through full-text evaluation, with 10 excluded due to unavailable survival data (*n* = 7), irrelevance of FAR (*n* = 2), and being a review article (*n* = 1). Finally, 10 articles comprising 4,704 cases were included in the meta-analysis [26–35] (Figure 1; Table 1).

**Table 1.** Basic characteristics of studies included in this meta-analysis.

Study	Year	Country	Sample size	Gender (M/F)	Age (years) Median(range)	Study design	Study period	Histology	TNM stage	Treatment	Cut-off value	Cut-off determination	Survival endpoints	Survival analysis	Follow-up (months) Median(range)	NOS score
Sun, F.	2018	China	702	582/120	59	Retrospective	2008–2013	CRC	I–III	Surgery	10.9	X-tile	OS	Multivariate	1–36	8
Chen, Q. G.	2019	China	507	284/223	≤60y: 257 >60 y: 250	Prospective	2011–2015	CRC	IV	Surgery + CRT	10.1	X-tile	OS, PFS	Univariate	1–36	9
Zhang, L.	2019	China	71	44/27	60	Retrospective	2015–2019	CRC	IV	Chemotherapy	10.63	ROC curve	PFS	Univariate	6.6(1.9–27.2)	7
Li, H.	2020	China	320	225/95	54	Retrospective	2009–2016	RC	II–III	nCRT + surgery	11.56	ROC curve	OS, PFS	Multivariate	1–60	7
Liao, Y. C.	2021	China	157	91/66	≤60y: 97 >60 y: 60	Retrospective	2010–2017	CRC	I–III	Surgery	9.26	X-tile	PFS	Multivariate	1–36	8
Ying, H. Q.	2021	China	1533	970/563	≤60y: 745 >60 y: 788	Retrospective	2013–2016	CRC	II–III	Surgery	10.87	X-tile	PFS	Multivariate	1–36	8
Xie, H.	2022	China	657	406/251	59(17–92)	Retrospective	2012–2014	CRC	I–IV	Surgery	12.0	X-tile	OS, PFS	Multivariate	63(1–80)	8
Zhao, X.	2023	China	200	115/85	61.5(24–81)	Retrospective	2010–2016	CRC	I–IV	Surgery	9.0	ROC curve	OS	Multivariate	1–100	7
Li, K.	2024	China	207	112/95	≤65y: 98 >65 y: 109	Retrospective	2017–2021	CRC	I–IV	Surgery	11.63	ROC curve	OS, PFS	Multivariate	45(25–62)	8
Yan, H.	2024	China	350	200/150	66(57–69)	Retrospective	2016–2018	CRC	II–III	Surgery	8.43	ROC curve	OS, PFS	Multivariate	1–60	8

CRC, colorectal cancer; RC, rectal cancer; CRT, chemoradiotherapy; nCRT, neoadjuvant chemoradiotherapy; ROC, receiver operating characteristic; OS, overall survival; PFS, progression-free survival; NOS, Newcastle–Ottawa Scale; M, male; F, female; TNM, tumor-node-metastasis.

### Study characteristics

Table 1 summarizes the basic characteristics of the included articles [26–35]. All eligible studies were conducted in China and published in English [26–35] between 2018 and 2024. Sample sizes ranged from 71 to 1,533 (median: 335). Nine studies were retrospective [26,28–35], and one was a prospective trial [27]. Nine studies included patients with CRC [26–28,30–35], while one focused on patients with rectal cancer [29]. Regarding cancer staging, three studies included patients with stages I–IV CRC [31–33], three focused on stages II–III cases [29,34,35] and two articles each enrolled patients with stages I–III [26,30] and IV [27,28] CRC. The FAR threshold ranged from 8.43 to 12, with a median of 10.75. Five articles utilized the receiver operating characteristic curve [28,29,32–34] to determine the FAR threshold, while five others applied the X-tile software [26,27,30,31,35]. Seven studies highlighted the significance of FAR in predicting OS [26,27,29,31–34] and eight provided data on the association between FAR and PFS [27–31,33–35]. Multivariate regression was used to derive HRs and 95% CIs in eight studies [26,29–35], while two adopted univariate regression [27,28]. NOS scores ranged from 7 to 9, indicating that all included articles were of high quality (Table 1).

### FAR and OS

**Figure 2.**
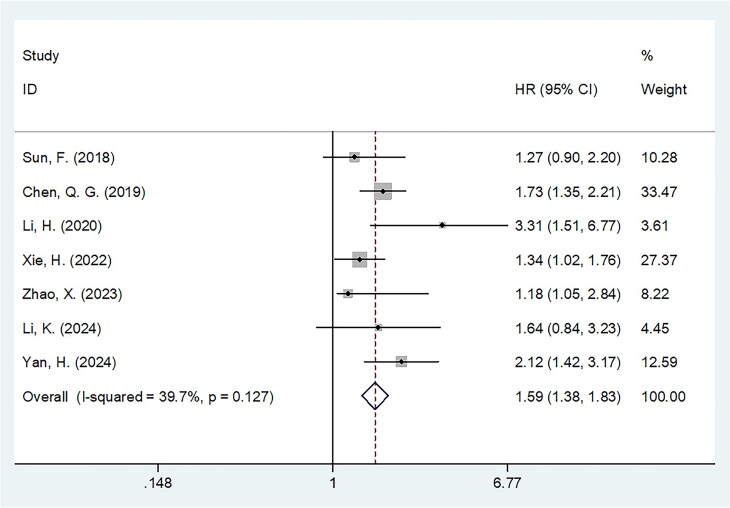
Forest plot of the association between FAR and OS in patients with CRC.

Seven studies involving 2,943 cases [26,27,29,31–34] evaluated the role of FAR in predicting OS. A fixed-effects model was used due to insignificant heterogeneity (*I*^2^ = 39.7%, *p* = 0.127). Elevated FAR was significantly associated with poor OS among patients with CRC (HR = 1.59, 95% CI = 1.38–1.83, *p* < 0.001; Figure 2; Table 2). Subgroup analysis revealed that high FAR remained a significant predictor of poor OS, regardless of study design, histology, treatment, FAR threshold, threshold determination method, and type of survival analysis (all *p* < 0.05, Table 2). Additionally, FAR was strongly associated with poor OS in subgroups with a sample size >300 (*p* < 0.001) and among patients with TNM stages II–III (*p* < 0.001) and IV (*p* < 0.001) (Table 2).

**Table 2.** Subgroup analysis of prognostic value of FAR for overall survival in patients with CRC.

Subgroups	No. of studies	No. of patients	Effects model	HR (95%CI)	*p*	Heterogeneity *I*^2^(%) PH	
Total	7	2943	Fixed	1.59(1.38–1.83)	<0.001	39.7	0.127
Sample size							
≤300	2	407	Fixed	1.33(0.89–1.98)	0.168	0	0.439
>300	5	2536	Random	1.68(1.32–2.14)	<0.001	52.8	0.076
Study design							
Prospective	1	507	–	1.73(1.35–2.21)	<0.001	–	–
Retrospective	6	2436	Fixed	1.52(1.28–1.81)	<0.001	46.0	0.099
Histology							
CRC	6	2623	Fixed	1.54(1.33–1.78)	<0.001	18.3	0.295
RC	1	320	–	3.31(1.56–7.01)	0.002	–	–
TNM stage							
I–III	1	702	–	1.27(0.81–1.98)	0.299	–	–
II–III	2	670	Fixed	2.34(1.64–3.34)	<0.001	5.0	0.305
I–IV	3	1500	Fixed	1.34(1.07–1.67)	0.012	0	0.740
IV	1	71	–	1.73(1.35–2.21)	<0.001	–	–
Treatment							
Surgery	5	2116	Fixed	1.45(1.21–1.74)	<0.001	18.2	0.299
Surgery + CRT/ nCRT + surgery	2	827	Random	2.16(1.18–3.97)	0.013	61.6	0.106
Cut–off value							
≤10.5	3	1057	Fixed	1.71(1.41–2.07)	<0.001	38.3	0.198
>10.5	4	1886	Fixed	1.45(1.17–1.79)	0.001	45.0	0.142
Cut–off determination							
X–tile	3	1866	Fixed	1.50(1.27–1.77)	<0.001	18.5	0.293
ROC curve	4	1077	Random	1.85(1.24–2.74)	0.002	50.0	0.112
Survival analysis							
Univariate	1	507	–	1.73(1.35–2.21)	<0.001	–	–
Multivariate	6	2436	Fixed	1.52(1.28–1.81)	<0.001	46.0	0.099

FAR, fibrinogen-to-albumin ratio; CRC, colorectal cancer; RC, rectal cancer; CRT, chemoradiotherapy; nCRT, neoadjuvant chemoradiotherapy; ROC, receiver operating characteristic.

### FAR and PFS

**Figure 3.**
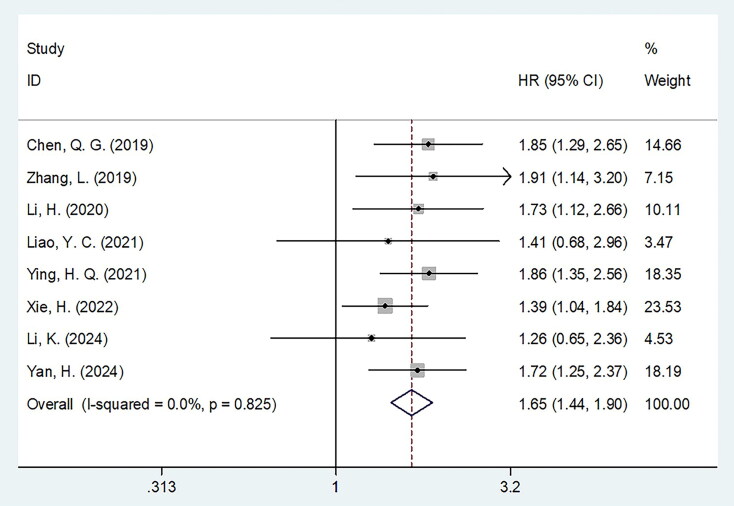
Forest plot of the association between FAR and PFS in patients with CRC.

Eight studies involving 3,802 patients [27–31,33–35] explored the relationship between FAR and PFS in CRC. A fixed-effects model was used due to insignificant heterogeneity (*I*^2^ = 0, *p* = 0.825). The pooled analysis showed that elevated FAR was significantly associated with worse PFS (HR = 1.65, 95% CI = 1.44–1.90, *p* < 0.001; Figure 3; Table 3). Subgroup analysis confirmed that elevated FAR was consistently correlated with worse PFS, regardless of study design, sample size, histology, treatment, FAR threshold, threshold determination method, or survival analysis type (all *p* < 0.05, Table 3). Moreover, subgroup analysis indicated that FAR was significantly correlated with poor PFS among patients with stages II–III, I–IV, and IV CRC (*p* < 0.05, Table 3).

**Table 3.** Subgroup analysis of prognostic value of FAR for progression-free survival in patients with CRC.

Subgroups	No. of studies	No. of patients	Effects model	HR (95%CI)	*p*	Heterogeneity *I*^2^ (%) PH	
Total	8	3802	Fixed	1.65(1.44–1.90)	<0.001	0	0.825
Sample size							
≤300	3	435	Fixed	1.58(1.11–2.24)	0.012	0	0.587
>300	5	3367	Fixed	1.67(1.44–1.94)	<0.001	0	0.654
Study design							
Prospective	1	507	–	1.85(1.29–2.65)	0.001	–	–
Retrospective	7	3295	Fixed	1.62(1.40–1.88)	<0.001	0	0.788
Histology							
CRC	7	3482	Fixed	1.65(1.42–1.90)	<0.001	0	0.737
RC	1	320	–	1.73(1.12–2.67)	0.013	–	–
TNM stage							
I–III	1	157	–	1.41(0.68–2.96)	0.358	–	–
II–III	3	2203	Fixed	1.77(1.45–2.17)	<0.001	0	0.940
I–IV	2	864	Fixed	1.36(1.05–1.77)	0.019	0	0.801
IV	2	578	Fixed	1.87(1.39–2.51)	<0.001	0	0.917
Treatment							
Surgery	5	2904	Fixed	1.58(1.34–1.87)	<0.001	0	0.628
Surgery + CRT/nCRT + surgery	2	827	Fixed	1.80(1.36–2.37)	<0.001	0	0.818
Chemotherapy	1	71	–	1.91(1.14–3.20)	0.014	–	–
Cut-off value							
≤10.5	3	1014	Fixed	1.74(1.38–2.18)	<0.001	0	0.812
>10.5	5	2788	Fixed	1.61(1.35–1.91)	<0.001	0	0.575
Cut-off determination							
X-tile	4	2854	Fixed	1.63(1.36–1.94)	<0.001	0	0.475
ROC curve	4	948	Fixed	1.69(1.36–2.11)	<0.001	0	0.798
Survival analysis							
Univariate	2	578	Fixed	1.87(1.39–2.51)	<0.001	0	0.917
Multivariate	6	3224	Fixed	1.60(1.37–1.87)	<0.001	0	0.740

FAR, fibrinogen-to-albumin ratio; CRC, colorectal cancer; RC, rectal cancer; CRT, chemoradiotherapy; nCRT, neoadjuvant chemoradiotherapy; ROC, receiver operating characteristic.

### FAR and CRC clinicopathological factors

**Figure 4.**
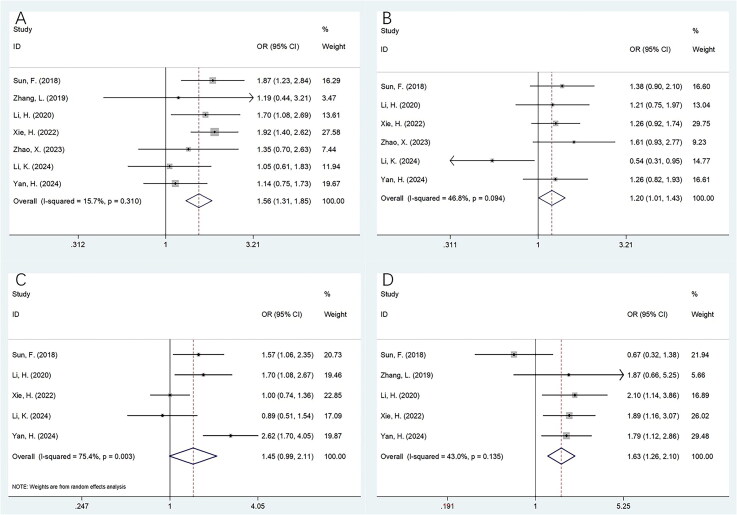
The relationship between FAR and clinicopathological features in patients with CRC. (A) Age (years) (≥60 vs < 60); (B) Gender (male vs female); (C) TNM stage (III–IV vs I–II); and (D) tumor differentiation (poor vs moderate/well).

**Figure 5.**
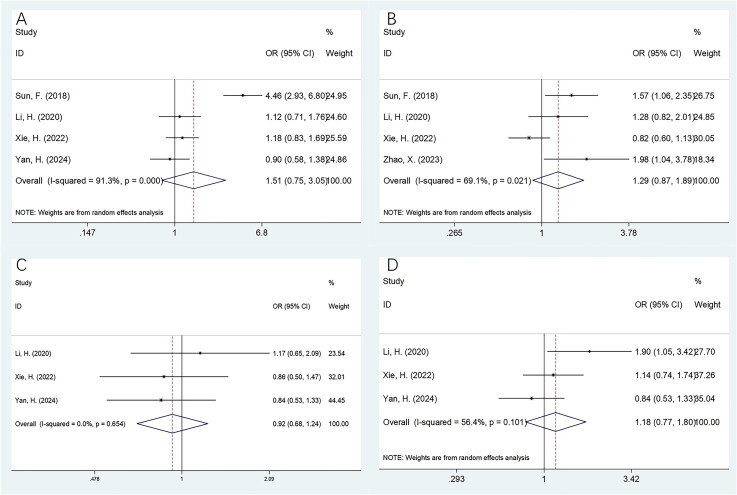
The relationship between FAR and clinicopathological features in patients with CRC. (A) Tumor size (cm) (>5 vs ≤5); (B) N stage (N1–N2 vs N0); (C) perineural invasion (yes vs no); and (D) vascular invasion (yes vs no).

Seven studies involving 2,507 cases provided data on the relationship between FAR and clinicopathological factors in CRC [26,28,29,31–34]. The pooled analysis revealed that elevated FAR was significantly associated with age ≥60 years (OR = 1.56, 95% CI = 1.31–1.85, *p* < 0.001), male sex (OR = 1.20, 95% CI = 1.01–1.43, *p* = 0.042), and poor tumor differentiation (OR = 1.63, 95% CI = 1.26–2.10, *p* < 0.001) (Figure 4; Table 4). However, no significant correlation was observed between FAR and TNM stage (OR = 1.45, 95% CI = 0.99–2.11, *p* = 0.055), tumor size (OR = 1.51, 95% CI = 0.75–3.05, *p* = 0.247), N stage (OR = 1.29, 95% CI = 0.87–1.89, *p* = 0.205), perineural invasion (OR = 0.92, 95% CI = 0.68–1.24, *p* = 0.595), or vascular invasion (OR = 1.18, 95% CI = 0.77–1.80, *p* = 0.449) (Figures 4 and 5; Table 4).

**Table 4.** The association between FAR and clinicopathological features in patients with CRC.

Variables	No. of studies	No. of patients	Effects model	OR (95%CI)	*p*	Heterogeneity *I*^2^ (%) PH	
Age (years) (≥60 vs <60)	7	2507	Fixed	1.56(1.31–1.85)	<0.001	15.7	0.310
Gender (male vs female)	6	2436	Fixed	1.20(1.01–1.43)	0.042	46.8	0.094
TNM stage (III–IV vs I–II)	5	2236	Random	1.45(0.99–2.11)	0.055	75.4	0.003
Tumor differentiation (poor vs moderate/well)	5	2100	Fixed	1.63(1.26–2.10)	<0.001	43.0	0.135
Tumor size (cm) (>5 vs ≤5)	4	2029	Random	1.51(0.75–3.05)	0.247	91.3	<0.001
N stage (N1–N2 vs N0)	4	1879	Random	1.29(0.87–1.89)	0.205	69.1	0.021
Perineural invasion (yes vs no)	3	1327	Fixed	0.92(0.68–1.24)	0.595	0	0.654
Vascular invasion (yes vs no)	3	1327	Random	1.18(0.77–1.80)	0.449	56.4	0.101

FAR, fibrinogen-to-albumin ratio; CRC, colorectal cancer.

### Sensitivity analysis

**Figure 6.**
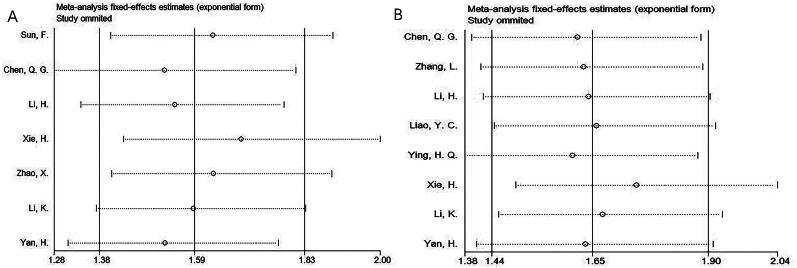
Sensitivity analysis. (A) OS and (B) PFS.

A sensitivity analysis was conducted to assess the stability of our meta-analysis outcomes. The combined analysis showed that the association of FAR with OS and PFS remained consistent, regardless of the exclusion of any individual study (Figure 6). These findings confirm the reliability and robustness of our results.

### Publication bias

**Figure 7.**
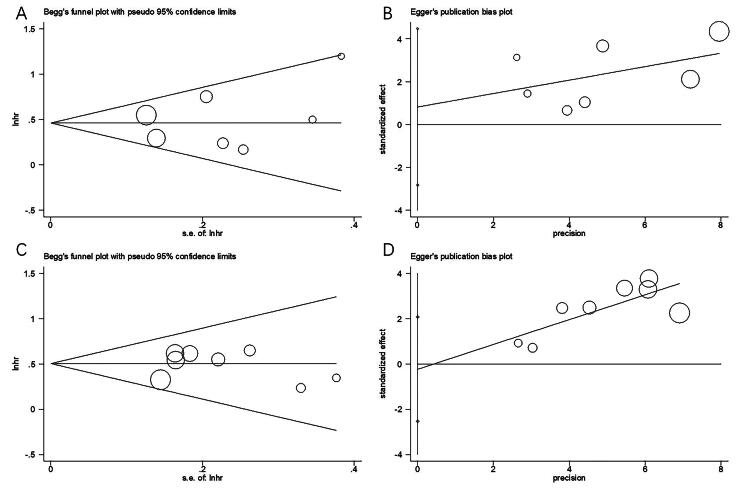
Publication bias test in this meta-analysis. (A) Begg’s test for OS, *p* = 0.548; (B) Egger’s test for OS, *p* = 0.589; (C) Begg’s test for PFS, *p* = 0.536; and (D) Egger’s test for PFS, *p* = 0.812.

Funnel plots, along with Begg’s and Egger’s tests, were used to examine potential publication bias. As shown in Figure 7, no significant publication bias was observed for OS (Begg’s test: *p* = 0.548; Egger’s test: *p* = 0.589) or PFS (Begg’s test: *p* = 0.536; Egger’s test: *p* = 0.812).

## Discussion

The prognostic value of FAR for predicting CRC prognosis has been explored in previous studies, yielding inconsistent results. This meta-analysis pooled data from 10 articles involving 4,704 cases [26–35], demonstrating that elevated FAR levels were significantly correlated with poor OS and PFS in CRC cases. The prognostic value of FAR remained significant across various subgroups. Moreover, a high FAR was significantly linked to age ≥60 years, male sex, and poor tumor differentiation in CRC. Publication bias tests and sensitivity analyses confirmed the robustness and reliability of these findings. Elevated FAR significantly predicted both short- and long-term prognostic outcomes in CRC. To our knowledge, this meta-analysis is the first to investigate the value of FAR for predicting CRC prognosis.

FAR is calculated using fibrinogen and albumin levels, with an elevated FAR resulting from increased fibrinogen and/or decreased albumin levels. The exact mechanisms underlying the prognostic value of FAR in CRC are not fully understood and are analyzed below. First, fibrinogen, a vital component of the coagulation system, plays a significant role in systemic inflammation. As an acute-phase glycoprotein, fibrinogen contributes to tumor invasion and progression by regulating coagulation, immune function, and inflammation [38]. When converted to fibrin with the help of thrombin, it forms a protective barrier surrounding cancer cells, promoting cancer cell survival and playing a crucial role in tumor development [39]. Second, serum albumin serves as a marker for both nutritional and immune status, with lower levels indicating poor nutrition and weakened immunity [40]. Hypoproteinemia has been linked to the release of inflammatory cytokines, such as interleukin (IL)-6 and tumor necrosis factor-alpha, which are associated with poor prognosis [41]. In addition, hypoalbuminemia is associated with impaired immune function due to macrophage activation [42]. IL-6 further exacerbates this by stimulating the liver to produce acute-phase proteins like C-reactive protein, increasing amino acid demand and depleting albumin levels. Therefore, FAR is a potential reliable prognostic marker for patients with CRC.

Notably, a high FAR value is associated with low albumin levels. Generally, in patients with cancer, albumin levels tend to drop more significantly during the middle and late stages of the disease, leading to hypoalbuminemia [43], which is indicative of malnutrition. Malnutrition in patients with cancer arises from various factors, including inflammation, disequilibrium between anabolic and catabolic processes, adverse effects of anti-cancer treatments, reduced food intake, and hormonal imbalances [44]. Decreased albumin levels can impair immune function, leading to a weaker response to cancer cells and promoting tumor growth [45]. Additionally, preoperative malnutrition is a common concern in patients with CRC and significantly impacts their postoperative recovery and outcomes [46]. Therefore, improving the nutritional status of patients with cancer during anti-cancer treatment is essential for enhancing their prognosis and overall well-being.

Recent meta-analyses have highlighted the prognostic value of FAR for predicting the prognosis of different cancers [47–50]. For instance, Zhang et al. performed a meta-analysis of 5,088 cases, concluding that elevated FAR was markedly associated with poor OS and worse disease-free survival (DFS) in malignant tumors [50]. Similarly, Sun et al. in a meta-analysis of 7,282 cases, demonstrated that high FAR predicted unfavorable outcomes, including OS, DFS, and PFS [49]. Additionally, Li et al. in a meta-analysis of 19 articles, found a strong association between higher FAR and poor cancer prognosis [47]. Our findings regarding the prognostic value of FAR in CRC are consistent with those observed in other cancers.

This study has certain limitations that should be acknowledged. First, all the included studies were conducted in China, necessitating validation of the prognostic value of FAR in CRC cases in other countries. Although no geographic restrictions were observed during article selection and only studies published in English were included, this geographic limitation may impact the generalizability of the findings. Second, the FAR threshold was inconsistent across the included articles, potentially introducing selection bias. Third, the majority of the included studies were retrospective in design, which may have contributed to heterogeneity in the results. To address these limitations, large-scale, multi-regional prospective studies are needed to validate our findings.

## Conclusions

In summary, elevated FAR is significantly associated with poor OS and PFS in patients with CRC. Additionally, higher FAR is strongly linked to older age and poor tumor differentiation in CRC.

## Supplementary Material

PRISMA_2020_checklist.docx

## Data Availability

The data that support the findings of this study are available from the corresponding author upon reasonable request.

## Abbreviations

FAR: Fibrinogen-to-albumin ratio
CRC: Colorectal cancer
HR: Hazard ratio
CI: Confidence interval
OS: Overall survival
PFS: Progression-free survival
OR: Odds ratio
PNI: Prognostic nutritional index
CONUT: Controlling nutritional status
GNRI: Geriatric nutritional risk index
PRISMA: Preferred Reporting Items for Systematic Reviews and Meta-Analyses
NOS: Newcastle-Ottawa Scale
ROC: Receiver operating characteristic
DFS: Disease-free survival
